# Sildenafil for Primary Prevention of Anthracycline-Induced Cardiac Toxicity: A Phase I/II Randomized Clinical Trial, SILDAT-TAHA6 Trial

**DOI:** 10.1155/2022/5681510

**Published:** 2022-03-27

**Authors:** Armin Attar, Masoumeh Heydari, Firoozeh Abtahi, Alireza Rezvani, Shirin Haghighat, Reza Vojdani, Mani Ramzi, Mehdi Dehghani, Mojtaba Karimi, Mohammad Kasaei, Shahdad Khosropanah, Mahmood Tabandeh

**Affiliations:** ^1^Department of Cardiovascular Medicine, School of Medicine, Shiraz University of Medical Sciences, Shiraz, Iran; ^2^Students' Research Committee, Shiraz University of Medical Sciences, Shiraz, Iran; ^3^Cardiovascular Research Center, Shiraz University of Medical Sciences, Shiraz, Iran; ^4^Hematology and Medical Oncology Department, Shiraz University of Medical Sciences, Shiraz, Iran; ^5^Hematology Research Centre, Shiraz University of Medical Sciences, Shiraz, Iran; ^6^Kowsar Hospital, Fars Heart Foundation, Shiraz, Iran

## Abstract

**Background:**

Previous animal studies have shown a protective effect of 5-phosphodiesterase inhibitors on cancer therapeutics-related cardiac dysfunction (CTRCD) of anthracyclines.

**Aim:**

The aim of this study was to evaluate the clinical effect of sildenafil on the primary prevention of CTRCD in human.

**Materials and Methods:**

In this randomized double-blind clinical trial, the primary end point was efficacy in preventing the reduction of left ventricular ejection fraction (LVEF). The intervention group patients received sildenafil at a dose of 25 milligrams twice a day before starting the chemotherapeutic regimen, and the control group received placebo. All the patients at baseline and after the 6-month follow-up underwent 4D and speckle-tracking echocardiography and cardiac MRI, accompanied by hs-troponin I and NT-Pro-BNP measurement.

**Results:**

Sixty patients were enrolled in this study, and data from 52 patients (24 patients in the intervention group and 28 patients in the control group) were used in the final analysis. Our findings showed that in the intervention and control groups, LVEF was dropped from 61.28 ± 7.36 to 51.57 ± 7.67 (difference (D) = −9.71 ± 11.95, *p*=0.003) and from 57.9 ± 7.29 to 50.2 ± 7.02% (*D* = −7.7 ± 5.93; *p*=0.001), respectively (between-group difference = −2.01%, *p*=0.26). CTRCD was detected in 11 patients in the control group (42.8%) and 10 in the intervention group (41.6%, *p*=0.51).

**Conclusion:**

Consumption of sildenafil for primary prevention of anthracycline-induced cardiac toxicity seems to be unbeneficial. This trial is registered with IRCT20180506039554N1.

## 1. Introduction

Chemotherapy is among the most common and useful therapeutic approaches for the wide spectrum of cancers. Chemotherapy-induced cardiotoxicity can be considered as a serious and life-threatening clinical complication that may limit the utilization of chemotherapy agents [1, 2]. Anthracyclines, as frequently used chemotherapeutic agents, include doxorubicin, daunorubicin, idarubicin, aclarubicin, and epirubicin. They are utilized for the treatment of lymphomas, cancers of breast, and also soft-tissue sarcomas [3, 4]. The most dangerous side effects of anthracyclines are cardiac toxicities. Cardiomyopathies related to the utilization of the anthracyclines include an immediate pericarditis-myocarditis syndrome, congestive heart failure, and late-onset cardiotoxicity that presents several years after treatment [5, 6].

The most prevalent cardiac related side effect of anthracyclines is cancer therapeutics-related cardiac dysfunction (CTRCD), which may advance to congestive heart failure [3–6]. It is reported that more than one-half of cancerous individuals who were under treatment with anthracyclines had a degree of cardiotoxicity several years after starting chemotherapy [7, 8]. However, only a small percentage of patients will develop overt heart failure. Several therapeutic modalities including treatment with beta blockers, renin-angiotensin-aldosterone system blockers, and even statins have been purposed for the prevention of CTRCD. However, none has been fully shown to be effective [1].

Phosphodiesterase-5 inhibitors (PDE5 inhibitors) are a class of drugs primarily introduced as cardioprotective agents and now is frequently used as a treatment of erectile dysfunction and pulmonary hypertension [9, 10]. These pharmacologic agents such as sildenafil, vardenafil, and tadalafil may affect the smooth muscle cells by providing relaxation, which leads to vasodilation [9, 10]. Several investigations have demonstrated that PDE5 inhibitors may have acceptable protective effect against myocardial ischemia/reperfusion injury, chemotherapy-induced cardiotoxicity, ischemic and diabetic cardiomyopathy, and the improvement of stem cell efficacy for myocardial repair [11, 12]. Two animal studies have investigated the effects of these medications on the preservation of the left ventricle ejection fraction (LVEF) with anthracycline therapy [13, 14]. Fisher and colleagues have shown that prophylactic treatment with sildenafil prevented apoptosis and left ventricular dysfunction in a chronic model of doxorubicin-induced cardiomyopathy in mice [13]. Koka and coworkers investigated the effect of tadalafil, a long-acting phosphodiesterase-5 (PDE-5) inhibitor, to protect against doxurubicin-induced cardiotoxicity. They noticed that tadalafil improved the left ventricular function and prevented cardiomyocyte apoptosis in doxurubicin-induced cardiomyopathy through mechanisms involving up-regulation of cGMP, PKG activity, and mitochondrial superoxide dismutase level without interfering with the chemotherapeutic benefits of doxurubicin [14].

## 2. Aim

To the extent of our knowledge, there are no human studies evaluating the protective effect of phosphodiesterase-5 (PDE-5) inhibitors on CTRCD. Therefore, the aim of this study was to determine the efficacy of sildenafil on the prevention of CTRCD among human subjects who had undergone chemotherapy with anthracycline agents.

## 3. Materials and Methods

### 3.1. Study Design and Participants

This placebo-controlled randomized double-blind clinical trial study was designed to enroll 60 patients in a 1 : 1 manner in the intervention and control groups, respectively. The primary end was efficacy in preventing the reduction of ejection fraction (EF).

We planned to enroll all the patients who received anthracyclines. Patients with any other concomitant diseases leading to consumption of beta blockers, ACE inhibitors, ARBs, or statins were excluded. In addition, patients with any history of ischemic heart disease, heart failure, significant congenital or acquired valvular heart disease, significant underlying disease such as severe and chronic renal failure, liver failure, and autoimmune disorders were excluded.

### 3.2. Sample Size Determination

According to the objectives and type of study and citing previous studies in this field [11, 12], taking into account the assumptions: 5% error and 80% power and the average difference of about 9 units with a standard deviation of 8 using the following formula:

(1) $$\begin{array}{c} n=\frac{2s^{2}{\left(Z_{1-\left(\alpha /2\right)}+Z_{1-B}\right)}^{2}}{{\left(\partial \right)}^{2}}. \end{array}$$

14 people in each group were estimated. Due to the length of the study and repeated measurements using the formula and a drop of 20%, the sample size is 17 in each group. We planned to enroll 30 in each group. In the abovementioned formula, the values of *z* are constant and equal to 97.5th percentile and 80th percentile of standard normal distribution. ∂ is the mean difference between the two groups, S2 is the combined variance of the two groups, and *P* is the amount of possible fall.

### 3.3. Randomization and Blinding

Randomization was done using the random permuted block system. This study was conducted in a double-blind way as the echocardiography, cardiac MRI operators, and the laboratory technicians were unaware of treatment assignment.

### 3.4. Intervention

The intervention group patients received sildenafil at a dose of 25 milligrams twice a day before starting the chemotherapeutic regimen, and the control group received a placebo with the same shape.

### 3.5. End Points

The primary end point was efficacy in preventing the reduction of ejection fraction (EF), and the secondary end point was efficacy for the prevention of CTRCD. CTRCD was defined based on the definition by the American Society of Echocardiography as either more than 10 percent drop in LVEF, 15% drop in GLS, LVEF drop below 50%, GLS drop below −19%, or pathological rise in the troponin level [15].

### 3.6. Ethical Considerations

This study conformed with the Declaration of Helsinki about working with human subjects and was approved by local ethics committee with an approval number of IR.SUMS.MED.REC.1393.61. It is also registered at IRCT with clinical trial registration number of IRCT20180506039554N1 (SILDAT-TAHA6 Trial). All the participant gave formal informed written consent before participating in the study. This study was monitored by local DSM, and initiation of phase II was based on their permission after evaluation of phase I.

### 3.7. Echocardiographic Study

For all patients, a baseline echocardiography study was done before the initiation of chemotherapy and a follow-up after 6 months when chemotherapy was finished. Three-dimensional transthoracic echocardiography (3D-TTE) was used to evaluate the patient's cardiac structure, LVEF, and global longitudinal strain (GLS). All patients were imaged in the left lateral decubitus position using the general electric E9 conventional echocardiography machine (GE, USA). The transducer was placed in the left midclavicular line in the 4^th^ to 5^th^ intercostal spaces, where the point of maximal impulses of the heart (PMI) was detected. A specified reader analyzed all the echocardiograms. LVEF was calculated by the 3D-TTE probe from the apical 4-chamber view using an automated 3D protocol method.

Speckle-tracking echocardiography was performed using the same machine; the displacement of the myocardial speckles in each spot was analyzed and tracked frame to frame. The longitudinal strain was assessed using automated functional imaging (AFI). The global longitudinal peak strain was automatically calculated as an averaged value of the peak longitudinal strain in all 3-image planes (apical 2- and 4-chamber and long-axis views).

The echocardiography was done by the authorized cardiologist who was blinded to all other parts of the study. The TTE was repeated six months later immediately after the completion of chemotherapy course.

### 3.8. Cardiac MRI Studies

The subjects underwent CMR imaging (1.5 T Magnetom® Avanto; Siemens Healthcare, Erlangen, Germany) at baseline and after 6 months. Serial contiguous short axis steady-state free precession (SSFP) cines were piloted from the vertical and horizontal long-axis images of the left and right ventricles (electrocardiogram *R* wave-gated, SSFP imaging (TrueFISP); temporal resolution, 40–50 ms; repetition time, 3.2 ms; echo time, 1.6 ms; flip angle, 60°; and slice thickness, 7 mm with 3-mm gap). Analysis of SSFP images was performed manually offline (Argus software; Siemens Healthcare) by a single blinded observer for the assessment of LVEF, LV end-diastolic volume (LVEDV), LV end-systolic volume (LVESV), and LVM. The LV basal short axis slice was identified as the image containing at least 50% of circumferential myocardium at the end diastole. Papillary muscles were included in the mass and excluded from volumetric analyses.

### 3.9. Serum Biomarker Measurement

All the patients underwent the measurement of highly sensitive troponin I and NT-Pro-BNP measurement at baseline before staring chemotherapy and after 6 months using enzyme-linked immunosorbent assay (ELISA) kits (bioassay technology laboratory, China).

### 3.10. Statistical Analysis

The analysis of the parametric data was expressed based on the mean and standard deviation. Qualitative and classified data were presented based on the number and percentage. Data were analyzed using SPSS (v.22. IBM Inc. IL). The normality Kolmogorov–Smirnov test was carried out to estimate whether continuous variables were normally distributed. Qualitative and classified data were presented based on the number and percentage and the univariate analysis on quantitative and qualitative data using independent and paired samples'*t*-test and chi-square tests. The *P* value less than 0.05 was considered significant.

## 4. Results

### 4.1. Patients' Demographic and Baseline Data

**Figure 1:**
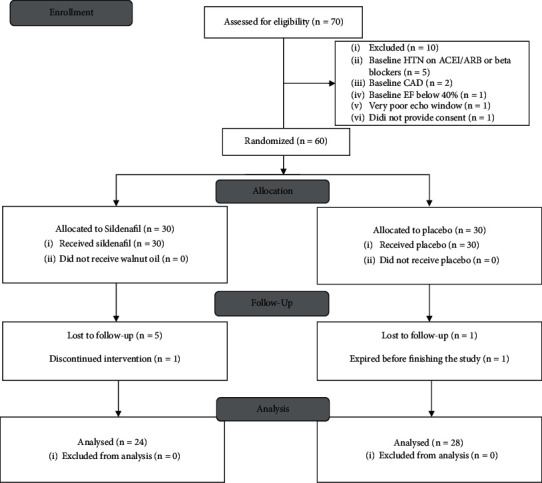
CONSORT 2010 flow diagram for this randomized, double-blind, placebo-controlled clinical trial of the effects of sildenafil on the prevention of anthracycline-induced cardiac toxicity.

The CONSORT flow diagram of the study is presented in Figure 1. From 70 patients screened for enrollment, 60 patients were finally enrolled and data from 52 patients were used at the final analysis (24 patients in the case group and 28 individuals in the control group). Baseline demographic data of the patients are presented in Table 1.

### 4.2. Echocardiography Studies

Baseline echocardiographic findings were not different between the two groups (Table 2). In the intervention and control groups, LVEF was dropped from 61.28 ± 7.36 to 51.57 ± 7.67 (difference (*D*) = −9.71 ± 11.95, *p*=0.003) and from 57.9 ± 7.29 to 50.2 ± 7.02% (*D* = −7.7 ± 5.93; *p*=0.001), respectively (between-group difference = −2.01%, *p*=0.26). Comparison of other echocardiographic findings showed the trends toward hazard with the intervention (Table 3).

Two patients in the control and none in the intervention group developed with diastolic dysfunction (*P*=0.57; Table 4).

### 4.3. Cardiac MRI Findings

Unfortunately, due to the urgency of starting the chemotherapies as soon as possible, none of the patients could perform their cardiac MRI before the initiation of anthracycline. Consequently, we have omitted the data of baseline cardiac MRI and only report the final MRI results. 24 patients (12 from each group) had a final cardiac MRI study. LVEF was 53.6 ± 4.6% and 53.7 ± 5.1% in the control and intervention groups, respectively (*P*=0.97). The mean LVEF was 52.1 ± 6.9% when assessed by 3D echo and 53.6 ± 4.9% when assessed by CMR (difference = 1.55 ± 1.92%, *P*=0.15), and there was a moderate correlation between MRI and echocardiographic findings (*r* = 0.55, *P*=0.008).

### 4.4. Serum Biomarker Level

All the patients' baseline troponin level was undetectable (<0.01 ng/ml). With chemotherapy, only one patient in each group developed with a troponin rise to a pathological level (>0.4 ng/ml), which did not show any significant difference (*P*=0.73). The baseline NT-Pro BNP level was 326 ± 33 and 299 ± 32 in the control and intervention groups, respectively (*p*=0.29). With chemotherapy, the NT-Pro BNP level did not rise to a pathological level in any patient (311 ± 35 and 298 ± 24 in the control and intervention groups, respectively; *p*=0.44).

### 4.5. Development of Cancer Therapeutics-Related Cardiac Dysfunction

In total, from 52 analyzed subjects, CTRCD was detected in 10 patients (41.6%) in the case group and 11 persons (42.8%) in the control group during the period of the study without a significant difference between the groups (*P*=0.51, Table 4).

## 5. Discussion

This study is the first clinical trial evaluating the effect of 5-phosphodiesterase inhibitors on the prevention of anthracycline-induced cardiac toxicity. Here, we have shown that LVEF did not reduce in patients receiving sildenafil less than the control group (between-group difference = −2.01%, *p*=0.26). These data raises warning that treatment with sildenafil not only has a protective effect on cardiotoxicity of anthracycline but alas there may be trends toward harm for these patients.

In our study, we did notice that treatment with sildenafil does not prevent the left ventricle from becoming dilated and reduction of LVEF. In fact, while preliminary results about some other interventions were encouraging, large clinical trials showed them to be futile. Kalay and colleagues evaluated the effect of 12.5 mg carvedilol on the primary prevention of CTRCD on 50 patients. They noticed that this intervention prevented 16.6% LVEF drop [16]. However, in a larger CECCY trial on 192 patients, there was no such effect and LVEF reduction was only 1.3% different between the groups, which was not significant [17]. The same results were noticed in the PRADA trial. While preliminary results were interesting, the results of that study only showed a 1.8% difference in the LVEF dropped by the treatment of metoral [18]. These controversies go back to some factors. First of all, our tools and techniques for the measurement of LVEF have improved. We used an automated 4D echocardiographic evaluation technique as well as cardiac MRI, which was not used in any other previous studies. Furthermore, the dosage of anthracyclines and patient's population enrolled were not similar in earlier studies and more recent ones. As our techniques of measurements and sample populations are becoming more precise, the results are more in favor of futility of such treatments.

In our study, there was no significant difference between the groups about the incidence of CTRCD. Several preliminary studies have shown that sildenafil, as a phosphodiesterase-5 inhibitor, may have an acceptable protective effect against myocardial ischemia/reperfusion injury, chemotherapy-induced cardiotoxicity, ischemic and diabetic cardiomyopathy, and the improvement of stem-cell efficacy for myocardial repair [12, 13, 19, 20]. However, larger more recent trials have shown contrary results. In a study conducted by Liu et al., it is mentioned that a treatment with sildenafil for 3 months in patients suffered from pulmonary hypertension, and heart failure with preserved ejection fraction cannot improve the cardiac function and structure [21]. In another report by Leung and colleagues, it was indicated that treatment with sildenafil for a period more than 6 months in adults suffering from muscular dystrophy and cardiomyopathy (ejection fraction ≤50%) may not improve the cardiac function [22]. That may happen for prevention of CTRCD. Previous animal studies have shown protective effects of 5-phosphodiesterase inhibitors on CTRCD. Fisher et al. in their study on 24 male mice in 4 treatment groups including saline, doxorubicin, sildenafil, and sildenafil plus doxorubicin reported that doxorubicin could cause a considerable increase in apoptosis, and disruption of mitochondrial membrane potential 12 in vitro [13]. They reported that sildenafil had a significant effect in the protection against cardiotoxicity resulting from doxorubicin [13]. Therefore, they reported that prophylactic treatment with sildenafil prevented apoptosis and left ventricular dysfunction in a chronic model of doxorubicin-induced cardiomyopathy [13]. Koka and coworkers investigated the effect of tadalafil, a long-acting phosphodiesterase-5 (PDE-5) inhibitor, to protect against doxurubicin-induced cardiotoxicity. They noticed that tadalafil improved the left ventricular function and prevented the cardiomyocyte apoptosis in doxurubicin-induced cardiomyopathy through mechanisms involving up-regulation of cGMP, PKG activity, and mitochondrial superoxide dismutase level without interfering with the chemotherapeutic benefits of doxurubicin [14]. However, our study, as the first clinical trial in the field, proved contrary in the human subjects.

Our study had some limitations. We observed a high number of cases of CTRCD. That was due to the definition used for defining CTRCD. Many studies just use the criteria of more than 10% LVEF drop. Considering only that criterion, only 28% of our patients in the control group could be defined as CTRCD. Furthermore, we used an automated 4D echocardiographic method to measure LVEF, which is the most accurate way for this assessment. While numerous studies only used only 2D echocardiography, we used a more sensitive method for monitoring the changes in the LVEF which enhanced our ability to detect the cases of CTRCD. Anthracycline-induced cardiotoxicity may occur after the completion of treatment, and its sign and symptoms can become apparent during several years after treatment with this chemotherapy agent in patients suffering from malignancies [5, 6]. It is important that in more than fifty percent of patients with a positive history of a cancer in childhood period that were treated with anthracyclines, there is the evidence of contractile abnormalities and dysfunction of the left ventricle in echocardiographic evaluation in adolescence [12, 13, 19, 20].

## 6. Conclusion

Consumption of sildenafil for primary prevention of anthracycline-induced cardiac toxicity seems to be unbeneficial. This finding once again clarifies that there is a large difference between the results from animal studies and human subjects; it is suggested that careful phase I trials should be done before the introduction of any new treatments.

## Figures and Tables

**Table 1:** Baseline demographic patients' data.

	Intervention group	Control group	*P* Value
*Baseline malignancy*			
Breast cancer	66.7%	50%	0.55
Lymphoma	25%	25%	0.62
Other cancers	12.5%	25%	0.52
Anthracycline dosage	361.5 ± 125.7	418.75 ± 76.05	0.24
Age (years)	42.6 ± 12.4	41.8 ± 16.8	0.32
Sex	66.7%	75%	0.48
*Baseline laboratory data*			
WBC count (n/dl)	8542 ± 4505	5112 ± 1398	0.06
Hemoglobin (mg/ml)	12.53 ± 1.06	12.65 ± 0.47	0.08
Platelet count (n/dl)	300571 ± 103542	247500 ± 60663	0.19
Serum creatinine .05 (mg/dl)	0.88 ± 0.13	1.01 ± 0.17	0.75
Asparate aminotransferase level (AST)	23.43 ± 9.51	21 ± 9.71	0.55
Alanine aminotransferase level (ALT)	25.86 ± 8.47	21.63 ± 19.47	0.36

**Table 2:** Baseline echocardiographic parameters.

Echocardiographic parameters	Groups	Mean ± SD	*P* Value
EF (echocardiography 4D)	Case	58.57 ± 6.70	0.88
	Control	56.72 ± 7.63	

GLS	Case	20.58 ± 1.28	0.39
	Control	20.87 ± 1.88	

LVEDD	Case	47 ± 7.1	0.2
	Control	47.5 ± 4.1	

LVESD	Case	28.42 ± 6.05	0.57
	Control	29.15 ± 4.68	

LVEDV	Case	48.14 ± 13.58	0.48
	Control	55 ± 23.53	

LVESV	Case	18.85 ± 5.95	0.34
	Control	23.52 ± 12.08	

TAPSE	Case	23 ± 3.7	0.48
	Control	22.92 ± 3.75	

S' RV	Case	0.11 ± 0.009	0.07
	Control	0.11 ± 0.02	

LVEF = left ventricular ejection fraction; GLS = global longitudinal strain; LVEDD = left ventricular end-diastolic diameter; LVESD = left ventricular end-diastolic diameter; LVEDV = left ventricular end-diastolic diameter; LVESV = left ventricular end-diastolic diameter; TAPSE = tricuspid annular plane systolic excursion; S' RV = S' right ventricle.

**Table 3:** Changes in echocardiographic parameters during the follow-up.

Parameters	Control group				Intervention group				Between groups difference	*P* value
	Baseline	After 6 month	Difference	*P* value	Baseline	After 6 month	Difference	*P* value		
LVEF (%)	57.9 ± 7.29	50.2 ± 7.02	−7.7 ± 5.9	0.001	61.28 ± 7.36	51.57 ± 7.67	−9.71 ± 11.95	0.003	−2.1 ± 3.4	0.26
GLS (%)	−20.87 ± 1.88	−19.17 ± 3.16	1.7 ± 2.94	0.031	−20.58 ± 1.28	−18.6 ± 2.4	1.98 ± 1.59	0.025	1.88 ± 6.63	0.71
LVEDD (mm)	47.5 ± 4.1	48.5 ± 5.4	1.05 ± 4.97	0.35	47 ± 7.1	50.57 ± 6.05	3.57 ± 5.22	0.12	2.52 ± 2.21	0.26
LVESD (mm)	29.15 ± 4.68	30.1 ± 5.2	0.95 ± 5.06	0.41	28.42 ± 6.05	31 ± 5.5	2.57 ± 3.7	0.12	1.62 ± 2.09	0.44
LVEDV (mm^3^)	55 ± 23.53	58.95 ± 25.39	3.4 ± 14.14	0.29	48.14 ± 13.58	59.14 ± 21.72	12 ± 16.7	0.11	8.6 ± 6.5	0.19
LVESV (mm^3^)	23.52 ± 12.08	29.15 ± 13.12	5.3 ± 8.1	0.008	18.85 ± 5.95	28.57 ± 9.67	10.42 ± 6.75	0.006	5.1 ± 3.4	0.14

LVEF = left ventricular ejection fraction; GLS = global longitudinal strain; LVEDD = left ventricular end-diastolic diameter; LVESD = left ventricular end-diastolic diameter; LVEDV = left ventricular end-diastolic diameter; LVESV = left ventricular end-diastolic diameter.

**Table 4:** Incidence of different criteria used for definition of cancer therapeutics-related cardiac dysfunction.

Parameters	Groups	Number (%)	*P* Value
LVEF decreased more than 10% absolute ejection fraction units	Case	8 (33)	0.404
	Control	8 (28.5)	

LVEF dropped to a level below 50%	Case	6 (25)	0.12
	Control	4 (14.3)	

GLS decrease was more than 15% of the baseline GLS value	Case	2 (8.3)	0.656
	Control	5 (17.8)	

Absolute number of GLS dropped to a level below 19%	Case	0	N/A
	Control	6 (21.4)	

Pathologic troponin rise	Case	2 (8.3)	0.733
	Control	1 (3.5)	

Total cases diagnosed as CTRCD^*∗*^	Case	10 (41.6)	0.31
	Control	12 (42.8)	

Development of diastolic dysfunction	Case	0	0.571
	Control	2	

LVEF = left ventricular ejection fraction; GLS = global longitudinal strain; CTRCD: cancer therapeutics-related cardiac dysfunction. ^*∗*^Some patients may develop with some of these criteria and CTRCD was defined by the presence of any of the mentioned criteria; consequently, the total number of CTRCD cases is less than the sums of each criterion.

## Data Availability

Data will be available by the corresponding author under reasonable request.
